# An *In Silico* Agent-Based Model Demonstrates Reelin Function in Directing Lamination of Neurons during Cortical Development

**DOI:** 10.1371/journal.pone.0110415

**Published:** 2014-10-21

**Authors:** James R. Caffrey, Barry D. Hughes, Joanne M. Britto, Kerry A. Landman

**Affiliations:** 1 Department of Mathematics and Statistics, University of Melbourne, Parkville, Victoria, Australia; 2 Florey Institute of Neuroscience and Mental Health, University of Melbourne, Parkville, Victoria, Australia; University of Memphis, United States of America

## Abstract

The characteristic six-layered appearance of the neocortex arises from the correct positioning of pyramidal neurons during development and alterations in this process can cause intellectual disabilities and developmental delay. Malformations in cortical development arise when neurons either fail to migrate properly from the germinal zones or fail to cease migration in the correct laminar position within the cortical plate. The Reelin signalling pathway is vital for correct neuronal positioning as loss of Reelin leads to a partially inverted cortex. The precise biological function of Reelin remains controversial and debate surrounds its role as a chemoattractant or stop signal for migrating neurons. To investigate this further we developed an *in silico* agent-based model of cortical layer formation. Using this model we tested four biologically plausible hypotheses for neuron motility and four biologically plausible hypotheses for the loss of neuron motility (conversion from migration). A matrix of 16 combinations of motility and conversion rules was applied against the known structure of mouse cortical layers in the wild-type cortex, the Reelin-null mutant, the Dab1-null mutant and a conditional Dab1 mutant. Using this approach, many combinations of motility and conversion mechanisms can be rejected. For example, the model does not support Reelin acting as a repelling or as a stopping signal. In contrast, the study lends very strong support to the notion that the glycoprotein Reelin acts as a chemoattractant for neurons. Furthermore, the most viable proposition for the conversion mechanism is one in which conversion is affected by a motile neuron sensing in the near vicinity neurons that have already converted. Therefore, this model helps elucidate the function of Reelin during neuronal migration and cortical development.

## Introduction

The neocortex is responsible for originating the complex motor, sensory and cognitive functions of the mammalian brain. Proper assembly of the six-layered neocortex requires the coordinated migration of pyramidal neurons into the embryonic cortical wall; a process that starts with generation and radial migration from the germinal zones and ends in cessation of movement in the cortical plate (CP). The progression of neurons leads to the formation of distinct layers with each sharing a similar morphology, pattern of connectivity and electrophysiology [1]–[3]. Each influx of neurons leads to an increase in the thickness of the cortex over time and abnormal neuronal migration is associated with a number of human brain disorders including lissencephaly, epilepsy and a spectrum of malformations of cortical development [4], [5].

**Figure 1:**
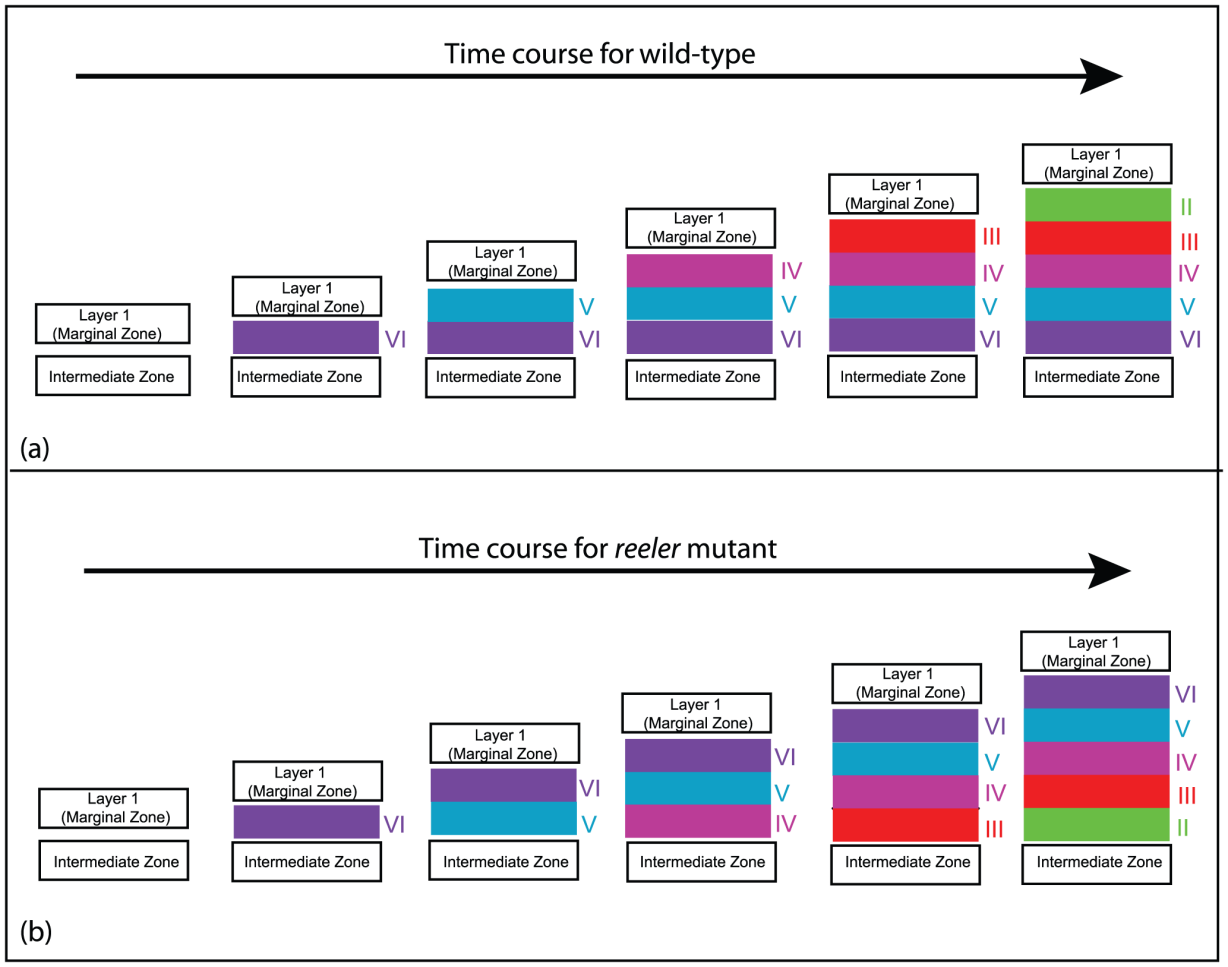
Schematic diagram of the “inside-out” cortical layering process in (a) wild-type and the “outside-in” cortical layering process in (b) *reeler* mutant development. The arrow indicates the progression of time. Layers VI, V, IV, III and II arise successively from neurons entering from the IZ. Although blending between layers occurs at the nominal boundaries, the existence of identifiable layers and the different spatial order of the layers in wild-type and *reeler* mutant is well established. For clarity the layers are shown to be distinct here. Only the layering (and not the preplate splitting) is shown in these figures.

Radial migration may account for how pyramidal neurons reach the CP, but it does not explain how these neurons form the characteristic six layers in the adult neocortex. For example, the lower (deep) layer V/VI neurons are the first to be born during corticogenesis whilst the upper (superficial) layer II/III neurons are the last to be born. This inside-out sequence of positioning occurs as upper layer neurons migrate past the predecessors and cease migration in the outermost region [6], [7], illustrated in Fig. 1(a). In spatial terms, the upper layer neurons “leap-frog” the predecessors and migrate the furthest from the germinal zones. The molecular cues underpinning such a highly orchestrated process are crucial for neuron placement in a stereotypic manner.

One such pathway, Reelin signalling, has proven crucial for this process as loss leads to disordered neuronal positioning in laminated structures of the brain including the neocortex, hippocampus and cerebellum [7]. During mouse cortical development, the absence of Reelin (*reeler* mutant) leads to two major phenotypes; first, abnormal splitting of the preplate, and second, partial inversion of cortical layers as shown in Fig. 1(b) [8]–[10]. The associated changes to the structure of the cortical layers are linked with impaired cortical functions [4], [11], [12]. The expression of the glycoprotein Reelin in cells residing in the marginal zone (MZ, referred to as layer 1) is well positioned to affect migration and placement of neurons in the CP. The Reelin-null mutant therefore provides a powerful tool in investigating the process of neuronal layer formation.

**Figure 2:**
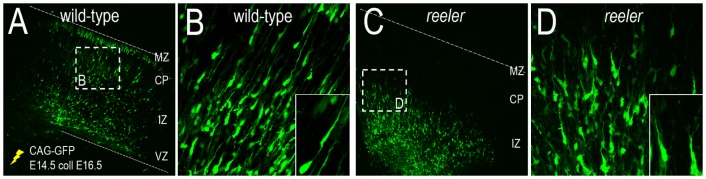
Distorted radial migration in the *reeler* mutant. (A) Confocal image of E16.5 wild-type cortex showing GFP-positive cells migrating from the ventricular zone (VZ) through the intermediate zone (IZ) and entering the cortical plate (CP), after electroporation at E14.5 (yellow). (B) High-power image of wild-type cortex shows the radial alignment of neurons migrating through the IZ with cells exhibiting a single process that extends towards the CP (inset). (C) Confocal image of E16.5 *reeler* cortex showing a restriction to the progression of migration. (D) High-power image of *reeler* cortex shows the loss of radial alignment, cells with multiple leading processes and loss of radial directionality (inset). In (A,C) a dashed line indicates the outer pial surface.

The role that Reelin plays during normal cortical development has been intensively studied and several alternative hypotheses have been proposed for how Reelin affects neuronal positioning into cortical layers. These include that Reelin acts as (i) a stopping signal [13], [14], (ii) a globally active chemoattractant thoughout the CP [15], [16], (iii) a locally active chemoattractant close to cortical layer I [15], [16] or (iv) a locally active repellant close to cortical layer I [16], [17]. A current model of inside-out lamination, “detach and go”, hypthesizes that multiple aspects of Reelin function may explain the phenotype exhibited in the *reeler* mutant [18]. In this model, migrating neurons convert from the glia-dependent locomotion mode of migration to somal translocation when detecting Reelin in the MZ. Recent evidence suggests that Reelin is required to polarize migrating neurons upon entry into the CP [19], which implies that the switch between locomotion and somal translocation may occur earlier as neurons enter the CP. Indeed, the altered migration in the *reeler* cortex is displayed as loss of directionality and movement of neurons in the intermediate zone (IZ) prior to entry into the CP (Fig. 2). This migratory phenotype can be rescued in the presence of exogenous Reelin [20], thus strengthening a role for Reelin when neurons migrate into CP.

To date there has been little exploration of the implications of competing models for examining the different hypotheses of Reelin function. A significant contribution to this debate is the qualitative discussion by Cooper [18] of two competing models. Our approach is to construct a mathematical model to determine which of the competing biological models best explains the multiple aspects of the lamination process. We use an agent-based model, often called a cellular automata (CA) model, to examine neuronal movement in the wild-type and Reelin-signalling mutants. Such a model is ideal for probing cell migration processes [21], such as the growth of a glioblastoma multiforme [22] and neural crest cell colonization of the developing enteric nervous system [23], [24].

Our agent-based model captures known and plausible behavior of the migrating neurons and the role of Reelin. In the model framework developed here, every agent represents a neuron. Four biologically plausible hypotheses for both neuron movement and conversion from a motile to immotile neuron are proposed. Local probabilistic rules are assigned to the model agents based on the biological hypothesis formulation. In this way, we mirror the biological formulation with a model formulation, with four movement hypotheses (a–d) and four conversion rules (i–iv). These rules are formulated in terms of the wild-type cortex and when modelling the Reelin mutants, the rules are modified accordingly. This approach provides a systematic study of four animal-based paradigms in terms of the model.

For each hypothesized role of Reelin, we test whether the model simulations give rise to the “inside-out” layering process of normal cortical development. From this testing, certain hypotheses are eliminated and others are strongly supported. Further elimination occurs in order to reproduce the reverse “outside-in” layering characteristic of the *reeler* mutant. In this systematic way, we eliminate more of the hypothesized mechanisms for the role of Reelin in neuron movement and conversion to an immotile movement.

Finally we test our model results against two other mutants, the Disabled-1 (*Dab1*) mutant [25] and *Dab1* conditional mutant [26], both of which exhibit abnormal cortical layering. Neurons in the *Dab1* mutant do not express Dab1 and therefore Reelin-signalling is blocked. This results in the “outside-in” layering similar to the *reeler* mutant [Fig. 1(b)]. In the *Dab1* conditional mutant, Dab1 expression is reduced after formation of the lower layers [26] and results in a loss of migration past the predecessors and misplacement of upper layer neurons.

The model results for all 16 combinations of motility and conversion rules against the wild-type and three Reelin mutants are determined. From these biological inferences are made to provide insight into the role of Reelin in the development of cortical layering. Furthermore, the model outcomes strongly implicate Reelin as a chemoattractant for neurons approaching the CP, while eliminating other possible outcomes. The findings of the mathematical model are discussed in the context of the current biological models.

## Methods

### Experimental methods

Experiments were undertaken with the approval of the Florey Neuroscience Institutes Animal Ethics Committee and conform to the Australian Code of Practice for the Care and Use of Animals for Scientific Purposes (7th Ed, 2004). Reln^r1/J^ (*reeler*) mice were genotyped as previously described [27]. Neurons were labelled with Green Fluorescent Protein (GFP) by *in utero* electroporation. The CAG-GFP plasmid [28] (Addgene plasmid 11150) was injected in the ventricle of E14.5 embryos using a glass micropipette and electroporated into cortical cells with five 50 ms square pulses at 40 V administered at 950 ms intervals (CUY21EDIT square-wave pulse generator). Embryos were allowed to develop for 48 h before tissue was fixed and processed for confocal microscopy imaging.

### Wild-type hypotheses: biological formulation

Pyramidal neurons migrating from the germinal zones undergo four phases of migration.

First, young neurons from the last cell division migrate along the radial glial fiber by locomotion. Second, the neurons convert to a transitory multipolar morphology within the subventricular zone (SVZ). Third, whilst within the IZ, neurons revert to a bipolar shape and move in an upward direction as observed in Fig. 2(a,b). Since this directional polarization is not as pronounced in the *reeler* mutant, Reelin is thought to be responsible for this directional bias. Finally, neurons migrate to the outer CP in a glial-independent manner referred to as somal translocation [18], [29], [30]. Reelin has been proposed to play a role at this later stage as neurons move towards a concentrated source in the MZ.

Although Reelin has been shown to affect neuronal proliferation and migration in the germinal zones [27], [31], the focus of our study is to examine the last two phases of neuronal migration through the IZ and entry into the CP. Four alternative roles for Reelin in neuron movement in the CP are hypothesized and investigated here.

1. *Reelin does not affect neuron movement*. The propensity of the neuron to move in a given direction and in the opposite direction is equal.
2. *Reelin acts as a global attractant*. Reelin is able to diffuse across the cortex and produce a chemoattractant gradient from bottom to top, inducing biased movement towards Layer I throughout the whole CP.
3. *Reelin acts as a local attractant near Layer I*. Reelin is detected by neurons only when the neurons are sufficiently close to Layer I, that is, within a sensing distance 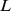. Beyond the sensing distance there is no upward bias, but within the sensing distance there is a bias towards Layer I.
4. *Reelin acts as a local repellant near Layer I*. Reelin is detected by neurons only when the neurons are sufficiently close to Layer I, that is, within a sensing distance 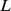. Beyond the sensing distance there is no vertical bias, but within the sensing distance a bias away from Layer I.

Two opposing biological models hypothesized are the “detach and stop” model [10], [32]–[34] and the “detach and go” model [18]. We incorporate aspects of these models in the movement rules above, and also by introducing another mechanism that changes the state of the neuron from active to passive with respect to its motility. A full comparison of the relationship between our model hypotheses and previous work is made in the Discussion section.

After a neuron moves from the IZ into the CP, we suggest that there is a mechanism which signals to a neuron to stop active movement and become part of the newly formed cortical layer. Reelin may or may not play a role in converting such an actively motile neuron to a passive neuron. A passive neuron is one that has stopped active migration. A passive neuron cannot initiate a move, but can undergo a translocation through interactions with an active motile neuron. Hence, once a neuron is passive it may or may not be in its final position.

Four possible mechanisms are hypothesized.

- *Reelin plays no role in the conversion of an active neuron to a passive neuron.* Conversion occurs globally throughout the cortical layer region.
- *Reelin plays no role in the conversion of an active neuron to a passive neuron.* Conversion occurs locally and depends on a neuron either sensing passive neuron from its current batch of neurons, or sensing neurons that make up older cortical layers.
- *Reelin acts a stop signal when neuron makes contact with Layer I.* In this case, conversion occurs instantaneously on contact with Layer 1.
- *Reelin acts a stop signal and there is local conversion (unaffected by the presence of Reelin).* This combines (ii) and (iii).

### Model overview

An agent represents a neuron. Two types of agents are considered. The first are actively *motile* agents and their movement is given by local rules governed by probabilities. These motile agents enter the IZ and eventually pass from the IZ into the CP. For a time, the agents retain their ability to move actively within the CP. At some time (governed by probabilities), the agent is no longer able to move actively, thereby becoming a *passive* agent. We call this a conversion mechanism from an active to a passive state. A passive agent cannot initiate a move, but can undergo a translocation through a movement event initiated by an active motile agent. In this way, eventually, all agents become passive and the cortical layer is complete.

Each cortical layer will consist of passive agents produced by a new batch of motile agents. Five batches of neuron agents enter the IZ in succession and eventually form five cortical layers.

### Wild-type hypotheses: modelling formulation

The four types of movement and four conversion hypotheses outlined above are translated into local agent rules and so are mirrored in rules (a–d) and (i–iv) below. These of course apply only to active motile agents.

An agent occupies a single site of the two-dimensional square lattice, with unit spacing between adjacent lattice sites. The lattice is oriented so that the lower horizontal boundary corresponds to the germinal zone, from which the agents representing neurons enter the IZ and progress to the cortical layer region. Agents enter the system from the lower horizontal boundary and move through the IZ and cross into the CP. The IZ has a fixed width (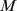 sites) and fixed height (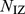 sites). The CP has same width (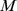 sites) but the height (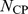) increases with time as groups of agents enter, move and at some time change state, that is, convert to passive agents. The topmost row, consisting of occupied sites in the CP, corresponds to cortical layer I. The details of the germinal zone, which lies below the IZ, and Layer I are not modeled here.

The neurons undergo negligible cell division within the IZ and CP, and consequently no agent division is included in the model.

#### Movement rules

**Figure 3:**
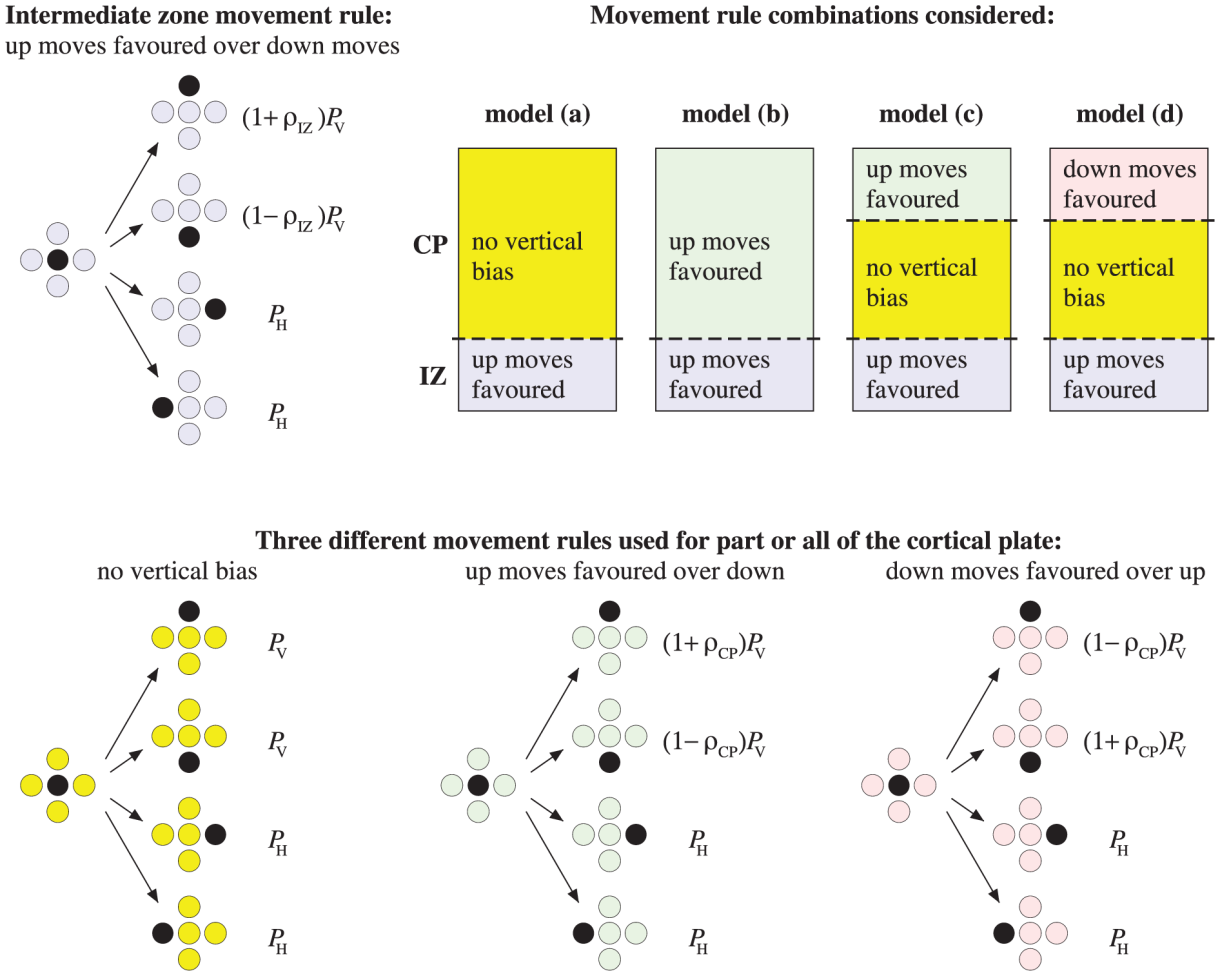
Schematic diagram showing examples of movement rules for motile neuron agents in the IZ and CP. Allowed moves are up, down, left, right. The probability that a vertical move is attempted (either up or down) is 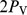, while the probability that a horizontal move is attempted (either left or right) is 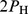, where 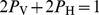. We take 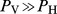, so that vertical moves in either direction are much more frequent that horizontal moves. For each movement rule illustrated, the motile agent (black) attempts one of the four possible moves with the probability shown. In the IZ, agents have a constant bias parameter 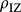. In the CP, the parameter 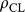 is used to quantify the extent of relative preference between up and down moves. Four movement mechanisms are considered: (a) random movement with no preferred vertical direction throughout the CP; (b) a spatially uniform upward bias (e.g., due to a constant chemotactic gradient) throughout the CP; (c) no preferred vertical direction close to the intermediate zone, but a short-range bias towards Layer I for neurons within a distance 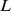 of Layer I (corresponding to a local attractive signal as Layer I is approached); (d) no preferred vertical direction close to the intermediate zone, but a short-range bias away from Layer I for neurons within a distance 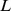 of Layer I (corresponding to a local repulsive signal as Layer I is approached).

Agents undertake random moves between nearest-neighbor sites (up, down, left or right). These are unit steps in four directions (two vertical and two horizontal) as shown in Fig. 3. Since neurons occupy a finite volume/area, each site can be occupied by at most one agent, making this an exclusion process [35].

A motile active agent, that is chosen to move, attempts to step to one of its four nearest neighbors with probabilities summarized in Fig. 3. The probability that a vertical move is attempted (either up or down) is 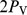, while the probability that a horizontal move is attempted (either left or right) is 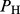, where 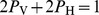. Here, vertical moves are more likely (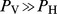). (We note, that the precise value of 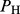 is not important as it effectively slows down the time scale of motility events relative to the time scale for conversion from active to passive agents.) For each movement rule illustrated, the motile agent (black) attempts one of the four possible moves with the probability shown.

In the IZ, neurons have a constant upward bias towards the CP. The bias is represented with a parameter 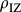.

In the CP, four movement mechanisms, corresponding to the biological formulation above, are considered.

- Random movement with no preferred vertical direction throughout the CP (independent of the presence of Reelin).
- A spatially uniform upward bias towards Layer I throughout the CP (dependent of the presence of Reelin).
- A short-range bias towards Layer I for neurons within a distance 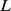 of Layer I, but no preferred vertical direction close to the IZ (dependent of the presence of Reelin).
- A short-range bias away from Layer I for neurons within a distance 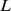 of Layer I, but no preferred vertical direction close to the IZ (dependent of the presence of Reelin).

Motility events that take an active agent into Layer 1, from the IZ back into the germinal layer or from the CP into the IZ are aborted.

*Agent interaction for movement events*: The local movement rules are affected by site occupancy constraint imposed by the exclusion process. In the IZ, if an (active motile) agent attempts to move onto a site occupied by another agent, the move is aborted. Since empty space is very limited in the CP, an alternative more flexible rule is implemented there. In the CP, if an agent attempts to move onto a site occupied by another agent (either active or passive), the two agents swap positions. However, since the CP is growing with time, we do not allow an agent to move into Layer 1. The formation of a new row, and therefore growth of the size of the CP, is related to the criteria used for crossing from the IZ to the CP. This is detailed below.

*Passive agents*: A passive agent cannot initiate a move, but can undergo a translocation through an event initiated by an active motile agent. In addition, passive agents from earlier layers may undergo movement events, but these can only occur due to interactions with an active movement event initiated by active younger agents. The only movement event allowed is swapping position with an active agent.

#### Conversion rules

**Figure 4:**
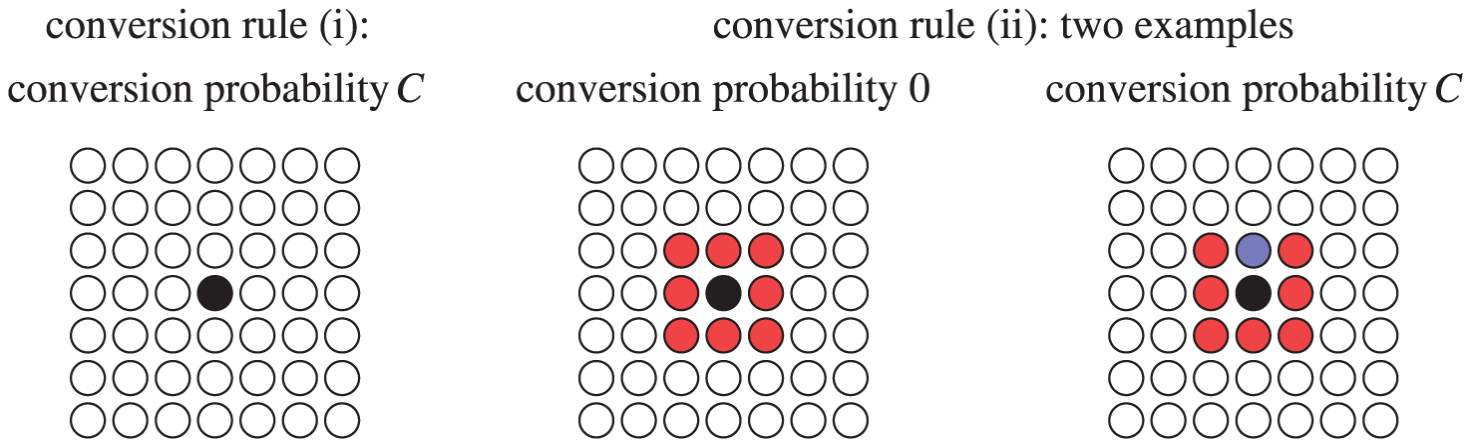
The conversion rules (i) and (ii) are illustrated. In each case the black disc represents an agent whose prospects for conversion are being assessed. Red discs correspond to active agents and blue discs to passive agents. White discs correspond to agents whose status (active or passive) is irrelevant. In rule (i), the black active agent has a probability 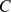 of converting, independent of the status of its neighbors. In rule (ii), the agent has probability 0 of converting if all of the eight agents in its Moore neighborhood (that is, the four nearest and four next nearest-neighbor sites) are active, while it has probability 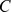 of converting if at least one of the eight agents in its Moore neighborhood is passive.

Agents can convert from an actively motile to a passively motile state only in the CP. The rate of conversion from the motile to immotile state is governed by a conversion probability 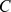, which is spatially uniform within a region where conversion is permitted, and may be constant in time or may vary with time. Four conversion mechanisms, corresponding to the biological formulation above, are considered. The mechanisms (i) and (ii) are illustrated in Fig. 4.

- Conversion occurs globally throughout the cortical layer region with constant probability 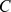 (independent of the presence of Reelin).
- Conversion occurs locally with constant probability 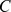 (independent of the presence of Reelin). It depends on an agent sensing either a passive agent from its current batch of agents, or sensing agents that make up previous cortical layers. Sensing is restricted to the identification of neighbors in the Moore neighborhood (8 neighbors).
- Conversion occurs when an agent is adjacent to Layer 1 (due to presence of Reelin) with probability unity.
- Conversion as in (ii) combined with (iii).

Note that the conversion of neurons giving rise to Layer VI under rule (ii) occurs through sensing Layer 1 and from passive agents from the current batch.

### Reelin signalling mutant models

*Reeler mutant*: In the absence of Reelin, there is evidence to suggest that neuron movement is less directed, as illustrated in Fig. 2(a,b). This corresponds to reducing the value of 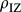. In addition, in the CP, the local movement and conversion rules must be modified to account for the absence of Reelin. In movement rules (b)–(d), we set 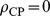, so there is no chemotactic bias. Movement rule (a) in unaffected for the *reeler* mutant.

*Dab1 mutant*: In the absence of Dab1, the Reelin signal cannot be received. Therefore, this could be tested using the same parameter values as for the *reeler* mutant, with the same outcomes. Here we explore an alternative possibility.

Previous studies have shown that changes in Dab1 protein levels affect the speed of neuronal migration in a cell-autonomous manner [36], [37]. Mutant Dab1 neurons move more slowly than wild-type neurons. Accordingly, we reduce the motility probability (now 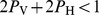), while keeping the movement bias and the conversion rate the same as in the wild-type.

*Dab1 conditional mutant*: We consider an *in silico* mutant where the neuron agents entering the IZ after a certain time have a reduced movement probability (reflecting the application of Cre recombinase and the generation of neurons without Dab1 protein). In our simulations, we make the last group of neuron agents, namely the group that will eventually form layer II as exhibiting the properties of a *Dab1* mutant, thus the conditional nature of this mutant. Therefore, all agents corresponding to VI-III have the properties of the wild-type model, while the last group has reduced movement probability, corresponding to the property of the *Dab1* mutant.

### Crossing from the IZ to CP and the formation of a new row in the CP

An entire row of agents enters synchronously into the CP as follows. Each agent is counted as it moves out of the IZ. These agents are placed in a queue or waiting room until there are sufficient agents to fill an entire row of the lattice (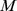). At this point the layered region grows by the addition of a full row of motile agents at the bottom of the CP, with all agents previously in this region being displaced one lattice spacing upwards. (Note that the model is robust to changing details of this boundary condition.)

### Algorithm updating

The position and state (whether actively or passively motile) of each agent are updated at discrete time steps. A batch of agents (labelled to identify the layer to which they belong) is introduced at the bottom IZ boundary. Let 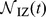, 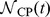 be the number of motile agents in the IZ and CP at time 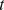, respectively. In each time step the following procedures occur.

1. Fill the bottom-most row of the IZ with agents (omit this if all agents that contribute to the layer have already entered the IZ).

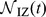
 independent random choices of agents in the IZ are made and they attempt to move, following rules outlined above.

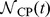
 independent random choices of motile agents in the CP are made and they attempt to move, following rules outlined above.

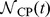
 independent random choices of motile agents in the CP are made and offered a chance convert to a passive agent, following rules outlined above. When the last motile agent in the CP converts to a passive immotile agent, the CP is complete and algorithm stops.

The agent-based model is simulated using Matlab version 7.11.

### Parameters

The IZ lattice dimensions are 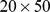 (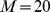 and 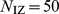). Two hundred agents are introduced for each of the five batches (to form the five CP layers). Therefore the average height of a layer 10 agents/neurons thick. Although the height of each cortical layer varies, this captures the correct order of magnitude, deduced from cell labelling illustrations [25], [38].

Each time step is of duration unity. In the IZ, a control value of the wild-type movement bias is 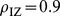, while the *reeler* mutant has a reduced bias of 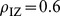.

The sensing radius in the movement rules (c,d) is taken as 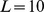 (Fig. 3 (c,d)). This value is the same scale as the final height of each layer. If 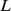 is too large, then it becomes a global parameter, while if too small, the effect is negligible.

Parameter values were varied by 10% to test the sensitivity of the model outcomes to parameter values. Additional testing was completed for other parameter values and results were qualitatively the same, and therefore not specific to the ones illustrated here.

## Results

The model results shown are the result of five successive inputs of neuron agents that pass through the IZ and enter the CP and eventually convert to immotile agents. The first layer to form is VI, then V, IV, III and the last layer is II, using the labelling convention applied to cortex development. This represents the process of corticogenesis where the oldest neurons are found in layer is VI and youngest neurons in layer II.

**Figure 5:**
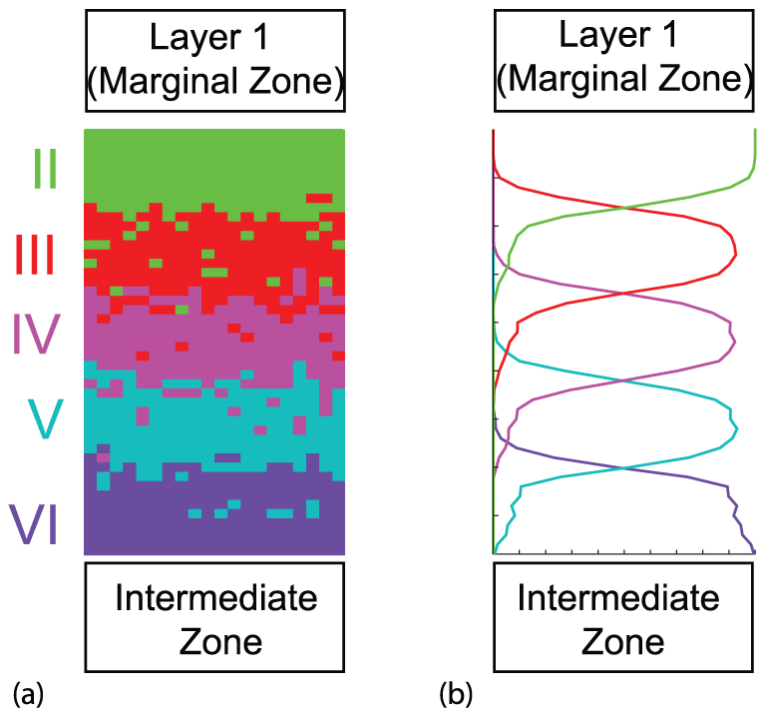
Wild-type cortical layering. (a) Model results from a single realization. (b) Model results averaged over 50 realizations. Vertical axis is distance and horizontal axis is agent density. Here the movement rule (b) and conversion rule (ii) were implemented. Here 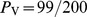, 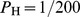, 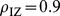, 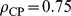, 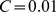 and 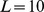. Similar results are observed with alternative mechanisms that successfully produce the wild-type phenotype indicated in Table 1.

**Figure 6:**
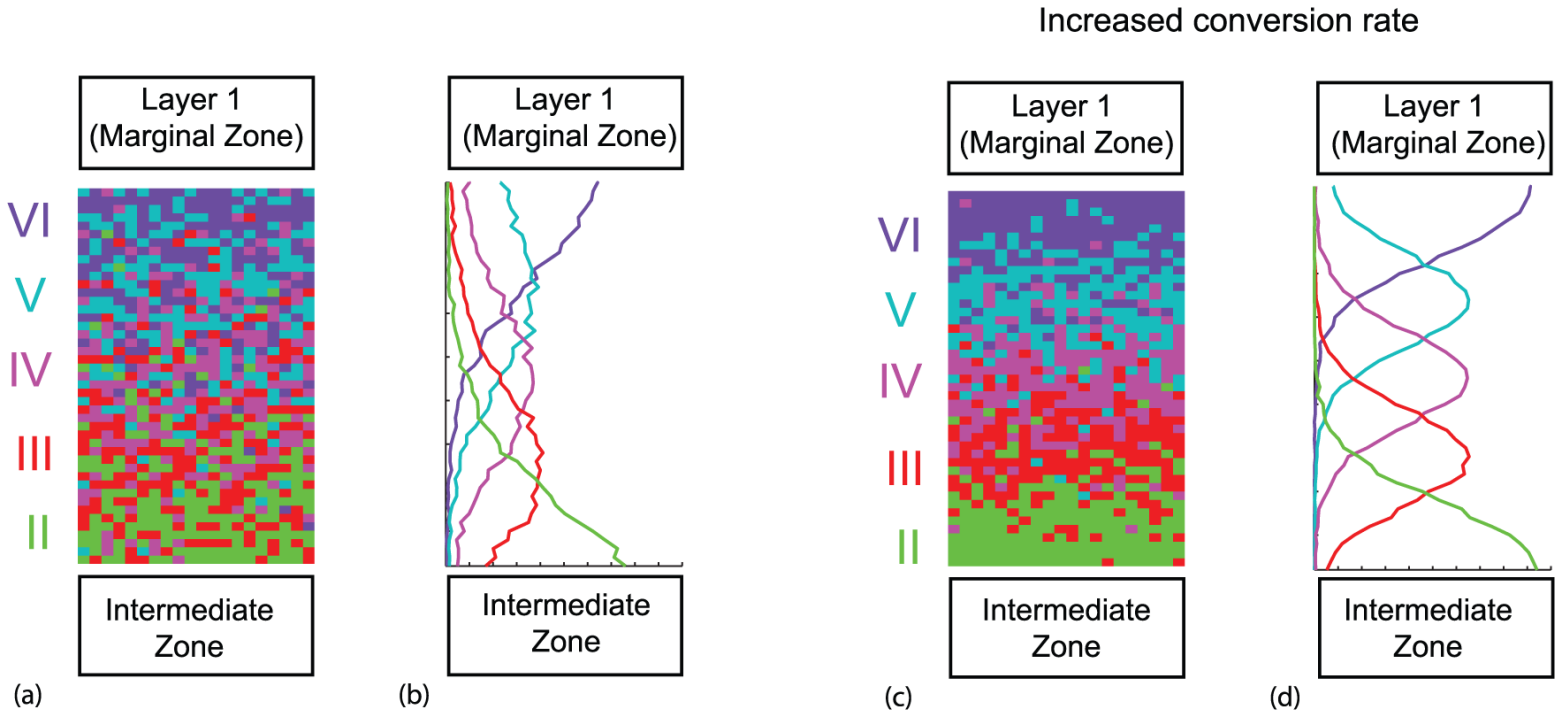
*Reeler* type cortical layering. (a, c) Model results from a single realization. (b,d) Model results averaged over 50 realizations. Vertical axis is distance and horizontal axis is agent density. Here the movement rule (b) and conversion rule (ii) were implemented, where the relevant mutant modifications were made. For (a, b) 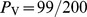, 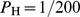, 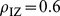, 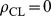, 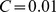 and 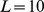, the same parameter values as for wild-type in Fig. 5. For (c,d) we used the increased conversion rate 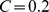. Similar results are observed with alternative mechanisms that successfully produce the *reeler* mutant phenotype indicated in Table 1.

**Figure 7:**
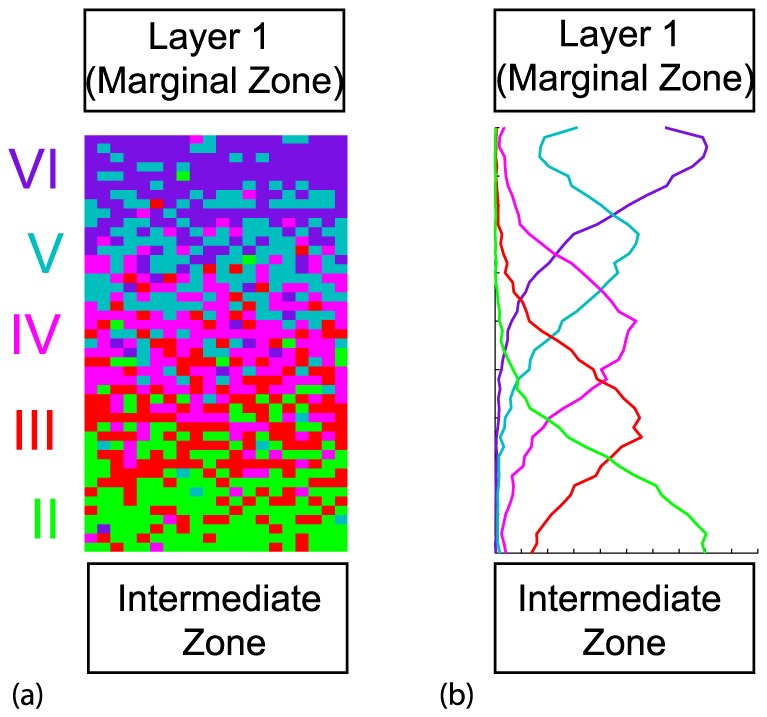
*Dab1* mutant type cortical layering. (a) Model results from a single realization. (b) Model results averaged over 50 realizations. Vertical axis is distance and horizontal axis is agent density. Here the movement rule (b) and conversion rule (ii) were implemented, where the relevant mutant modifications were made. Here: 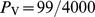, 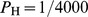, 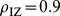, 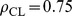, 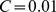 and 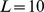. Similar results are observed with alternative mechanisms that successfully produce the *Dab1* mutant phenotype indicated in Table 1.

**Figure 8:**
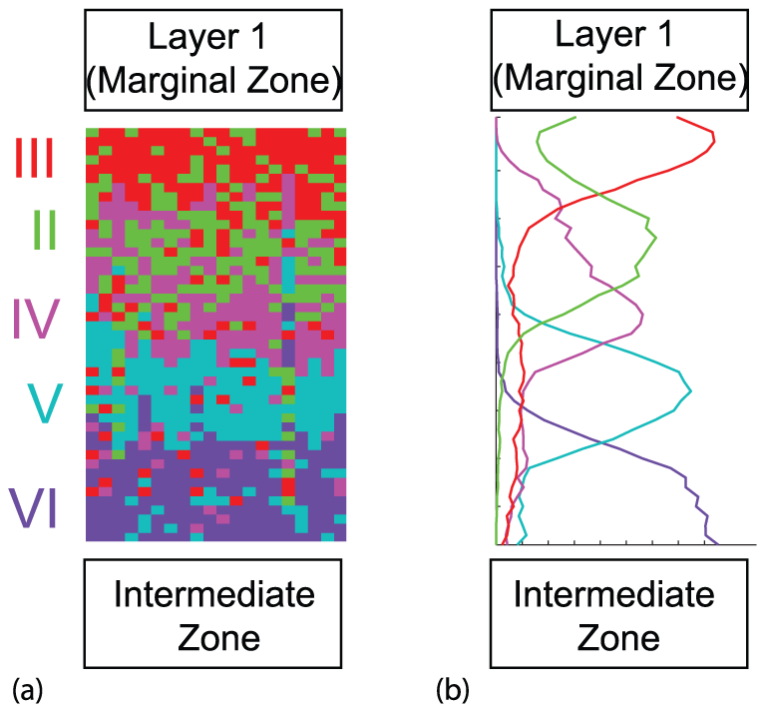
*Dab1* conditional mutant cortical layering, affecting only layer II. (a) Model results from a single realization. (b) Model results averaged over 50 realizations. Vertical axis is distance and horizontal axis is agent density. Here the movement rule (b) and conversion rule (ii) were implemented, where the relevant mutant modifications were made. Similar results are observed with alternative mechanisms that successfully produce the *Dab1* conditional mutant phenotype indicated in Table 1. For layers VI to III, 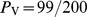, 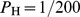, while for layer II 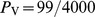, 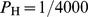. Also 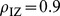, 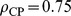, 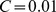 and 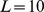.

For a fixed set of parameter values, each model simulation produces different results since the model is governed by probabilities. Averaging over many simulations (50 here) and averaging over each row produces an estimate of the average density as a function of density within the cortex. A single realization of the agent-based model, together with the row-averaged results over 50 simulations, are illustrated in Figs. 5–8.

We test four rules for agent movement and four rules for agent conversion from an active to a passive agent. Computational simulations are carried out for each of the 16 pairings to ascertain which of the pairings reproduce normal cortical layering pattern and various mutant patterns. Different mechanisms predict clear differences in outcomes, which are compared to biological findings.

### Wild-type model generates “inside-out” layering

For a single pair of neuron movement and conversion rules (movement (b) and conversion (ii)), the model generates a cortical layer structure as illustrated in Fig. 5. In a single simulation [Fig. 5(a)], five distinct layers are readily visible with the oldest (VI) at the bottom and the youngest (II) at the top. As anticipated, there is some mixing between the neuron groups at the boundary between layers.

When 50 such realizations are averaged, a plot of position versus agent density is obtained [Fig. 5(b)]. These figures clearly show the “inside-out” cortical layering observed in the wild-type cortex [7] and represented schematically in Fig. 1(a). Hence, the agent-based model is able to reproduce the normal cortical layering for this pair of hypotheses.

The results for all pairs are summarized in the appropriate ‘WT’ columns in Table 1. The results in Fig. 5 are typical of only some of the 16 pairs of rules. They cannot be produced with many other pairings. We see that all conversion types (i)–(iv) together with movement rules (b) and (c) produce the cortex layering observed in the wild-type cortex. Hence, the results imply that Reelin is either a global or local attractant to Layer 1 (marginal zone). In addition, the results strongly suggest that Reelin cannot act as a repellent. Furthermore, it seems unlikely that the Reelin does not affect neuron movement, unless conversion only occurs adjacent to Layer 1 (stop signal (iii)). For that combination of mechanisms, eventually randomly moving agents will find themselves adjacent to Layer 1 and will convert and become immotile, resulting in the normal “inside-out” layering.

**Table 1:** Phenotypes that result from the 16 combinations of the four motility mechanisms (a)–(d) and the four conversion rules (i)–(iv).

Conversion Rule Used in Cortical Layer	Movement Rule in Cortical Layer															
	(a) Unbiased random				(b) Bias due to				(c) Layer 1 attracting				(d) Layer 1 repelling			
	movement				chemoattractant				sensing bias				sensing bias			
	WT	R	D	D^C^	WT	R	D	D^C^	WT	R	D	D^C^	WT	R	D	D^C^
(i) Global at random	X	√	X	X	✓	√	√	X	✓	√	√	X	X	√	X	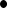
(ii) Local at random	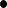	√	X	√	✓	√	√	√	✓	√	√	√	X	√	X	√
(iii) Stop signal	√	**X**	**X**	**X**	✓	**X**	**X**	**X**	✓	**X**	**X**	**X**	√	**X**	**X**	**X**
(iv) Stop signal + local	√	√	X	X	✓	√	√	X	✓	√	√	X	√	√	X	√

Combinations that successfully reproduce a phenotype over a range parameter values are shown with tick marks (√). The two columns in which the tick marks are shown in an enlarged size correspond to especially informative predictions. Combinations that sometimes reproduce the experimental observations are shown with bullets (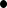). Failure to reproduce the experimental observation is shown with a cross (X). The row in which the crosses are shown in an enlarged size corresponds to especially informative predictions. The phenotypes considered are: WT  =  Wild-type, R  =  *reeler* mutant, D  =  *Dab1* mutant and D^C^  =  *Dab1* conditional mutant. Note, for the Reelin signalling mutants, mechanism (iv) converts to (ii).

### *Reeler* mutant model generates “outside-in” layering

When considering the associated mutant version, the parameters associated with the Reelin are modified as discussed in the Methods section.

For movement rule (b) and conversion rule (ii), the model produces a cortical layer structure with layers, but now the youngest layer (II) is at the bottom and the oldest layer (VI) is at the top [Fig. 6]. This is the reverse layering observed in the *reeler* cortex [8]–[10] and represented schematically in Fig. 1(b). With the Fig. 5 conversion probability value, substantial mixing between agents belonging to the different layers is observed in both the individual and average simulation in Fig. 6(a,b). However, as the conversion rate increases, the layers become more distinct, as illustrated in Fig. 6(c,d). There is some discrepancy in the literature as to how distinct the layer boundaries actually are in the *reeler* mutant [39]. Regardless, we have demonstrated that the layer ordering is preserved (reversed from the wild-type). Furthermore, we have shown that the layering distinctness is modified when conversion rate is varied. This also occurs if the ratio 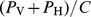 decreases (i.e. holding 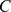 fixed and decreasing the movement probability).

The results across the 16 mechanism pairs are summarized in the appropriate ‘R’ columns in Table 1. We conclude that the *reeler* mutant cannot be reproduced when the conversion mechanism is only type (iii) (Reelin acting as a stop signal at Layer 1, thereby inactive in a *reeler* mutant). In this case the agents are always motile and can never convert to the immotile state. No layers form and the agents from the five batches become well-mixed. Therefore the hypothesis that Reelin acts only as a stop signal should be rejected. The *reeler* mutant is successfully produced for all four movement mechanisms as long as the conversion is either global or local (since (iv) converts to (ii) for the Reelin signalling mutants).

The wild-type analysis gave restrictions on the movement mechanism alone, while the *reeler* provides restrictions only on the conversion mechanism. When we combine the results of these two cases, there are now limitations on both the movement and conversion mechanisms. Our model results strongly suggest that Reelin acts as a chemoattractant, and not as a stopping agent.

### *Dab1* mutant model generates “outside-in” layering

Slowing the movement probabilities, while keeping the conversion probability a constant, can result in reverse layering profile observed in the *Dab1* mutant [25], just like the *reeler* mutant and shown schematically in Fig. 1(b). This in illustrated in Figure 7 for movement (b) and conversion rule (ii) Visual comparison shows that the patterning demonstrated in Fig. 7 is more distinct than those in Fig. 6(a,b), while less district than those in Fig. 6(c,d) for the parameter choice illustrated. This result is consistent with our previous discussion since we are modifying the ratio 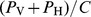.

The results across the 16 mechanism pairs are summarized in the appropriate ‘D’ columns in Table 1. In particular, two of the columns in Table 1 associated with the *Dab1* mutant (namely mechanisms (a) and (d), where Reelin plays no role in movement or is a repellent) cannot produce the reverse patterning observed in these mutants.

We deduce that the *Dab1* mutant model results reinforce the conclusion that Reelin must be acting as a chemoattractant.

### *Dab1* conditional mutant changes placement of affected layer

Finally, we consider the *Dab1* conditional mutant Fig. 8. All neurons corresponding to VI–III have the properties of the wild-type neurons, and therefore as the different groups of neurons enter the cortical layer region, the first four layers will look just like a wild-type. However, the last group of neurons making up layer II is effectively a *Dab1* mutant with reduced probability of movement in the IZ and CP. These agents form a layer between layer IV and III, and therefore do not accumulate adjacent to Layer I, as occurs in the wild-type. Therefore, the final arrangement has Layers VI, V, IV in the same arrangement as in a wild-type, but there is reverse ordering for the last two layers. Therefore, only the group of treated neurons that act like *Dab1* mutant neurons, give rise to a misplacement of layering, as observed in experiments [26]. The results for all pairs are summarized in the appropriate 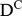 columns in Table 1.

## Discussion

Disruption of the Reelin gene in humans results in lissencephaly (smooth brain) with clinical systems of severe ataxia, epilepsy and cognitive delay [4], [5]. It is therefore imperative to understand how Reelin directs the placement of neurons during corticogenesis. Until now, the only exploration of the implications of competing biological models has been qualitative. Here we present a mathematical model of cortical development. This allows for a measured and systematic quantitative analysis.

We have developed an agent-based model that describes the formation of neuron cortical layers. Such discrete models capture individual-level properties of a biological system that reflect the stochasticity and non-uniformity observed in experiments. We formulated several plausible alternative influences of Reelin to represent the different hypotheses found in the literature. These provided stochastic rules for both neuron movement and their conversion from an actively motile to a passively motile neuron, which have been quantitatively expressed and explored. In total 16 combinations of the model were tested.

The results from the wild-type and three Reelin signalling mutants presented in Table 1 are combined together in Table 2 to summarize and highlight our findings. There are only two paired mechanisms (shown with a green tick mark in the table) that reproduce the four phenotypical cortical layering patterns observed in wild-type and three Reelin signalling mutants. These two mechanisms are that Reelin is a chemoattractant (either global or local to Layer 1) and the conversion from an actively motile to a passive neuron is determined locally, driven from a signal from already passive neurons.

**Table 2:** Conclusions drawn from the results in Table 1 from evidence over all four phenotypes.

Conversion Rule Used in Cortical Layer	Movement Rule in Cortical Layer			
	(a) Unbiased random	(b) Bias due to	(c) Layer 1 attracting	(d) Layer 1 repelling
	movement	chemoattractant	sensing Reelin bias	sensing bias
(i) Global at random	X	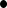	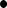	X
(ii) Local at random	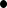	✓	✓	X
(iii) Stop signal	**X**	**X**	**X**	**X**
(iv) Stop signal + local	X	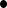	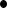	X

A combination that correctly reproduces all four phenotypes over a range of parameter values are shown with tick marks (✓). Combinations that fail for two or more phenotypes are shown with a cross (X). The row in which the crosses are shown in an enlarged size corresponds to especially informative predictions. A combination that fails on one phenotype is shown with a bullet (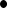). Note, for the Reelin signalling mutants, mechanism (iv) converts to (ii).

Furthermore, nine paired mechanisms are definitively inappropriate and do not produce the phenotypes. The remaining five fail on one phenotype. By considering the seven cases marked with a tick or bullet (the latter indicating that only one phenotype was not produced correctly), we can be more definite about the movement rule than the conversion rule. A movement bias is needed. It is possible the conversion is random, but the simulation results show it is more likely that the conversion is driven by a signal from already immotile neurons.

Our model helps clarify aspects of the ongoing debate on the role of Reelin in the formation of cortical layers. In the models used in this study, the greatest contribution for layer formation is that (i) repulsion in the marginal zone is not appropriate to layer formation and (ii) chemotactic bias towards the marginal zone is required. This could be across the whole cortical layer, or within a certain sensing distance of the marginal zone. A sensing distance could occur by various means. For example, it could be that Reelin may only diffuse across a certain distance from the marginal zone. Alternatively, it may be that neurons can only detect Reelin when the concentration is above a certain threshold, and therefore is not detectable near the IZ zone for the younger layer formations. This may be relevant given a second source of Reelin is present in Layer V neurons at later stages of corticogenesis [41], a time when CP thickness prevents incoming neurons sensing the Reelin source in the MZ. The model clarifies the importance of the neurons having bias to move towards the marginal zone, and therefore supports the proposition that Reelin acts as a chemoattractant.

We now relate our conclusions from the mathematical model to the purely qualitative discussion by Cooper [18] of the two models “detach and stop” and “detach and go”. Cooper makes the following postulates for both models: neurons attached to radial glia move along these glial guides towards the top of the CP (‘locomotion’) and if Reelin is encountered, neurons on radial glia detach from them. The “detach and stop” model [10], [32]–[34] asserts that the only motility is glia guided. In this model, normal layering develops when neurons migrating on glia meet Reelin and detach, ceasing movement, thickening the CP and pushing the Reelin source in layer 1 further away from the IZ. Neurons introduced subsequently migrate along the glia past the neurons that have detached most recently, encounter Reelin, and then detach closer to the edge of the CP than the previous layer. In the absence of Reelin, detachment does not occur, ‘traffic jams’ occur on the glial guides, and layering becomes partially inverted as exhibited in the *reeler* mutant. Cooper finds several problems with this model, including evidence of migration in the CP that is not associated with glial guides.

This evidence motivates Cooper's proposing a modified model, which he describes (perhaps slightly misleadingly) as “detach and go”. For this model, he introduces an additional motility mechanism where neurons extend a process towards a Reelin source and subsequently pull the cell body towards the Reelin source by somal translocation. In the early stages of development, before glial guides have formed, there is somal translocation if Reelin is present at the top of the CP, but later waves of neurons from the IZ attach to radial glia, migrate first along glial guides, detach from the glia on detecting Reelin, and then undergo somal translocation. Cooper does not explain how somal translocation ends, or how neurons introduced later translocate further than earlier ones that have already reached the edge of the CP.

Our modeling addresses some aspects not explicitly covered by Cooper's discussion. We include mechanisms for loss of motility, and we allow motile neurons to displace immotile ones while respecting local volume conservation constraints. Cooper makes no statements concerning the relative speed of glial-guided migration and somal translocation. This implies that two of our mechanisms would fit within his qualitative perspective. These are our motility mechanism (b), or a blend of mechanisms (b) and (c) with different strength biases towards layer 1 for neurons close to layer 1 or far from layer 1. In summary, this reaffirms the role of Reelin as a source of chemoattractant.

There are elements of a neuronal change of state in the “detach and stop” and “detach and go” models. However, our introduction of an explicit change of state from an active to passive motile neuron allows us to explore further possibilities. One possibility is that Reelin acts as a stop signal at Layer 1 — however this could not reproduce the layering phenotype observed in the *reeler* mutant (there are non-apparent differences in our stopping mechanism to those discussed in Cooper [18]). Indeed if Reelin's role was only as a stopping agent, then in our model distinct layers would not form. The result is a well-mixed system with all neuron batches evenly spread throughout the cortex. In contrast, our quantitative work suggests that a local signal is required to convert a motile neuron into a neuron fated to be part of layer. The signal relies on the neuron sensing an immotile neuron from its own layer or older cortical layers. This mechanism was also postulated by Zubler et al. [40] in the context of modelling multiple aspects of normal cortical development, including cell proliferation, migration and branching processes. Since this idea arose in two independent models, it would be valuable if experiments could be devised to test this hypothesis.

Our work also relates to observations by Franco [26] on conditional mutants. The levels of Dab1 protein within a cell can affect the speed of neuronal migration in a cell-autonomous manner [36], [37]. We propose that the *Dab1* conditional mutant has a reduced motility, thus affecting the somal translocation step in normal development. Furthermore, our work suggests reasons for the variation in boundary distinctness between layers in the *reeler* mutants as observed by Dekimoto and colleagues [39]. The distinctness can be enhanced by increasing the conversion rate or by decreasing the motility. Furthermore, this was confirmed when slowing of movement probabilities of the *Dab1* neurons/agents in our model this affected the distinctness of inverted cortex produced there.

We have presented a simplified version of neuron migration in the agent-based model to represent cortical development. For example, the morphology of cells is not modelled [40]; rather any differences between the cells in the WT and mutants are lumped into motility parameters. Nonetheless, many of the key hypotheses of Reelin function in currency have been analyzed, as well as proposing some variations to these. In summary, the model does not support Reelin acting as a repelling or as a stopping signal. In contrast, the study lends very strong support to the notion that the glycoprotein Reelin acts as a chemoattractant for neurons approaching the CP. This is also supported by the presence of the additional source of Reelin in Layer V, to aid the directional movement of the incoming neurons in the expanding cortex [41]. We also suggest that a neurons ability to actively migrate may be affected by sensing neurons that have already ceased active migration.

The agent-based model contributes to the current debate on the role of Reelin during cortical development. Indeed, the ease of manipulating the *in silico* model means that we can test elements of cortical development not available through biological experimentation.
